# The potential of working hypotheses for deductive exploratory research

**DOI:** 10.1007/s11135-020-01072-9

**Published:** 2020-12-08

**Authors:** Mattia Casula, Nandhini Rangarajan, Patricia Shields

**Affiliations:** 1 Department of Political and Social Sciences, University of Bologna, Strada Maggiore 45, 40125 Bologna, Italy; 2 Texas State University, San Marcos, TX USA

**Keywords:** Exploratory research, Working hypothesis, Deductive qualitative research, Pragmatism

## Abstract

While hypotheses frame explanatory studies and provide guidance for measurement and statistical tests, deductive, exploratory research does not have a framing device like the hypothesis. To this purpose, this article examines the landscape of deductive, exploratory research and offers the *working hypothesis* as a flexible, useful framework that can guide and bring coherence across the steps in the research process. The working hypothesis conceptual framework is introduced, placed in a philosophical context, defined, and applied to public administration and comparative public policy. Doing so, this article explains: the philosophical underpinning of exploratory, deductive research; how the working hypothesis informs the methodologies and evidence collection of deductive, explorative research; the nature of micro-conceptual frameworks for deductive exploratory research; and, how the *working hypothesis* informs data analysis when exploratory research is deductive.

## Introduction

Exploratory research is generally considered to be inductive and qualitative (Stebbins 2001). Exploratory qualitative studies adopting an inductive approach do not lend themselves to a priori theorizing and building upon prior bodies of knowledge (Reiter 2013; Bryman 2004 as cited in Pearse 2019). Juxtaposed against quantitative studies that employ deductive confirmatory approaches, exploratory qualitative research is often criticized for lack of methodological rigor and tentativeness in results (Thomas and Magilvy 2011). This paper focuses on the neglected topic of deductive, exploratory research and proposes working hypotheses as a useful framework for these studies.

To emphasize that certain types of applied research lend themselves more easily to deductive approaches, to address the downsides of exploratory qualitative research, and to ensure qualitative rigor in exploratory research, a significant body of work on deductive qualitative approaches has emerged (see for example, Gilgun 2005, 2015; Hyde 2000; Pearse 2019). According to Gilgun (2015, p. 3) the use of conceptual frameworks derived from comprehensive reviews of literature and a priori theorizing were common practices in qualitative research prior to the publication of Glaser and Strauss’s (1967) *The Discovery of Grounded Theory*. Gilgun (2015) coined the terms Deductive Qualitative Analysis (DQA) to arrive at some sort of “middle-ground” such that the benefits of a priori theorizing (structure) and allowing room for new theory to emerge (flexibility) are reaped simultaneously. According to Gilgun (2015, p. 14) “in DQA, the initial conceptual framework and hypotheses are preliminary. The purpose of DQA is to come up with a better theory than researchers had constructed at the outset (Gilgun 2005, 2009). Indeed, the production of new, more useful hypotheses is the goal of DQA”.

DQA provides greater level of structure for both the experienced and novice qualitative researcher (see for example Pearse 2019; Gilgun 2005). According to Gilgun (2015, p. 4) “conceptual frameworks are the sources of hypotheses and sensitizing concepts”. Sensitizing concepts frame the exploratory research process and guide the researcher’s data collection and reporting efforts. Pearse (2019) discusses the usefulness for deductive thematic analysis and pattern matching to help guide DQA in business research. Gilgun (2005) discusses the usefulness of DQA for family research.

Given these rationales for DQA in exploratory research, the overarching purpose of this paper is to contribute to that growing corpus of work on deductive qualitative research. This paper is specifically aimed at guiding novice researchers and student scholars to the *working hypothesis* as a useful a priori framing tool. The applicability of the *working hypothesis* as a tool that provides more structure during the design and implementation phases of exploratory research is discussed in detail. Examples of research projects in public administration that use the working hypothesis as a framing tool for deductive exploratory research are provided.

In the next section, we introduce the three types of research purposes. Second, we examine the nature of the exploratory research purpose. Third, we provide a definition of working hypothesis. Fourth, we explore the philosophical roots of methodology to see where exploratory research fits. Fifth, we connect the discussion to the dominant research approaches (quantitative, qualitative and mixed methods) to see where deductive exploratory research fits. Sixth, we examine the nature of theory and the role of the hypothesis in theory. We contrast formal hypotheses and working hypotheses. Seven, we provide examples of student and scholarly work that illustrates how working hypotheses are developed and operationalized. Lastly, this paper synthesizes previous discussion with concluding remarks.

## Three types of research purposes

The literature identifies three basic types of research purposes—explanation, description and exploration (Babbie 2007; Adler and Clark 2008; Strydom 2013; Shields and Whetsell 2017). Research purposes are similar to research questions; however, they focus on project goals or aims instead of questions.

Explanatory research answers the “why” question (Babbie 2007, pp. 89–90), by explaining “why things are the way they are”, and by looking “for causes and reasons” (Adler and Clark 2008, p. 14). Explanatory research is closely tied to hypothesis testing. Theory is tested using deductive reasoning, which goes from the general to the specific (Hyde 2000, p. 83). Hypotheses provide a frame for explanatory research connecting the research purpose to other parts of the research process (variable construction, choice of data, statistical tests). They help provide alignment or coherence across stages in the research process and provide ways to critique the strengths and weakness of the study. For example, were the hypotheses grounded in the appropriate arguments and evidence in the literature? Are the concepts imbedded in the hypotheses appropriately measured? Was the best statistical test used? When the analysis is complete (hypothesis is tested), the results generally answer the research question (the evidence supported or failed to support the hypothesis) (Shields and Rangarajan 2013).

Descriptive research addresses the “What” question and is not primarily concerned with causes (Strydom 2013; Shields and Tajalli 2006). It lies at the “midpoint of the knowledge continuum” (Grinnell 2001, p. 248) between exploration and explanation. Descriptive research is used in both quantitative and qualitative research. A field researcher might want to “have a more highly developed idea of social phenomena” (Strydom 2013, p. 154) and develop thick descriptions using inductive logic. In science, categorization and classification systems such as the periodic table of chemistry or the taxonomies of biology inform descriptive research. These baseline classification systems are a type of theorizing and allow researchers to answer questions like “what kind” of plants and animals inhabit a forest. The answer to this question would usually be displayed in graphs and frequency distributions. This is also the data presentation system used in the social sciences (Ritchie and Lewis 2003; Strydom 2013). For example, if a scholar asked, what are the needs of homeless people? A quantitative approach would include a survey that incorporated a “needs” classification system (preferably based on a literature review). The data would be displayed as frequency distributions or as charts. Description can also be guided by inductive reasoning, which draws “inferences from specific observable phenomena to general rules or knowledge expansion” (Worster 2013, p. 448). Theory and hypotheses are generated using inductive reasoning, which begins with data and the intention of making sense of it by theorizing. Inductive descriptive approaches would use a qualitative, naturalistic design (open ended interview questions with the homeless population). The data could provide a thick description of the homeless context. For deductive descriptive research, categories, serve a purpose similar to hypotheses for explanatory research. If developed with thought and a connection to the literature, categories can serve as a framework that inform measurement, link to data collection mechanisms and to data analysis. Like hypotheses they can provide horizontal coherence across the steps in the research process.

Table 1 demonstrated these connections for deductive, descriptive and explanatory research. The arrow at the top emphasizes the horizontal or across the research process view we emphasize. This article makes the case that the working hypothesis can serve the same purpose as the hypothesis for deductive, explanatory research and categories for deductive descriptive research. The cells for exploratory research are filled in with question marks.

**Table 1:** Connecting research purpose and frameworks for deductive inquiry


Type of purpose	Micro-conceptual framework	Methodology	Data analysis	Primary philosophical underpinning
Explanatory	Formal hypotheses	Quantitative, experimental design, survey, time series, existing data	Inferential statistics	Positivism
Descriptive	Categories	Quantitative, survey, content analysis	Simple descriptive statistics	Positivism
**Exploratory**	**??? Q2**	**??? Q3**	**??? Q4**	**??? Q1**

The remainder of this paper focuses on exploratory research and the answers to questions found in the table:

1. What is the philosophical underpinning of exploratory, deductive research?
2. What is the Micro-conceptual framework for deductive exploratory research? [*As is clear from the article title we introduce the working hypothesis as the answer*.]
3. How does the *working hypothesis* inform the methodologies and evidence collection of deductive exploratory research?
4. How does the *working hypothesis* inform data analysis of deductive exploratory research?

## The nature of exploratory research purpose

Explorers enter the unknown to discover something new. The process can be fraught with struggle and surprises. Effective explorers creatively resolve unexpected problems. While we typically think of explorers as pioneers or mountain climbers, exploration is very much linked to the experience and intention of the explorer. Babies explore as they take their first steps. The exploratory purpose resonates with these insights. Exploratory research, like reconnaissance, is a type of inquiry that is in the preliminary or early stages (Babbie 2007). It is associated with discovery, creativity and serendipity (Stebbins 2001). But the person doing the discovery, also defines the activity or claims the act of exploration. It “typically occurs when a researcher examines a new interest or when the subject of study itself is relatively new” (Babbie 2007, p. 88). Hence, exploration has an open character that emphasizes “flexibility, pragmatism, and the particular, biographically specific interests of an investigator” (Maanen et al. 2001, p. v). These three purposes form a type of hierarchy. An area of inquiry is initially *explored*. This early work lays the ground for, *description* which in turn becomes the basis for *explanation*. Quantitative, explanatory studies dominate contemporary high impact journals (Twining et al. 2017).

Stebbins (2001) makes the point that exploration is often seen as something like a poor stepsister to confirmatory or hypothesis testing research. He has a problem with this because we live in a changing world and what is settled today will very likely be unsettled in the near future and in need of exploration. Further, exploratory research “generates initial insights into the nature of an issue and develops questions to be investigated by more extensive studies” (Marlow 2005, p. 334). Exploration is widely applicable because all research topics were once “new.” Further, all research topics have the possibility of “innovation” or ongoing “newness”. Exploratory research may be appropriate to establish whether a phenomenon exists (Strydom 2013). The point here, of course, is that the exploratory purpose is far from trivial.

Stebbins’ *Exploratory Research in the Social Sciences* (2001), is the only book devoted to the nature of exploratory research as a form of social science inquiry. He views it as a “broad-ranging, purposive, systematic prearranged undertaking designed to maximize the discovery of generalizations leading to description and understanding of an area of social or psychological life” (p. 3). It is science conducted in a way distinct from confirmation. According to Stebbins (2001, p. 6) the goal is discovery of potential generalizations, which can become future hypotheses and eventually theories that emerge from the data. He focuses on inductive logic (which stimulates creativity) and qualitative methods. He does not want exploratory research limited to the restrictive formulas and models he finds in confirmatory research. He links exploratory research to Glaser and Strauss’s (1967) flexible, immersive, Grounded Theory. Strydom’s (2013) analysis of contemporary social work research methods books echoes Stebbins’ (2001) position. Stebbins’s book is an important contribution, but it limits the potential scope of this flexible and versatile research purpose. If we accepted his conclusion, we would delete the “Exploratory” row from Table 1.

Note that explanatory research can yield new questions, which lead to exploration. Inquiry is a *process* where inductive and deductive activities can occur simultaneously or in a back and forth manner, particularly as the literature is reviewed and the research design emerges.^1^ Strict typologies such as explanation, description and exploration or inductive/deductive can obscures these larger connections and processes. We draw insight from Dewey’s (1896) vision of inquiry as depicted in his seminal “Reflex Arc” article. He notes that “stimulus” and “response” like other dualities (inductive/deductive) exist within a larger unifying system. Yet the terms have value. “We need not abandon terms like stimulus and response, so long as we remember that they are attached to events based upon their function in a wider dynamic context, one that includes interests and aims” (Hildebrand 2008, p. 16). So too, in methodology typologies such as deductive/inductive capture useful distinctions with practical value and are widely used in the methodology literature.

We argue that there is a role for exploratory, deductive, and confirmatory research. We maintain all types of research logics and methods should be in the toolbox of exploratory research. First, as stated above, it makes no sense on its face to identify an extremely flexible purpose that is idiosyncratic to the researcher and then basically restrict its use to qualitative, inductive, non-confirmatory methods. Second, Stebbins’s (2001) work focused on social science ignoring the policy sciences. Exploratory research can be ideal for immediate practical problems faced by policy makers, who could find a framework of some kind useful. Third, deductive, exploratory research is more intentionally connected to previous research. Some kind of initial framing device is located or designed using the literature. This may be very important for new scholars who are developing research skills and exploring their field and profession. Stebbins’s insights are most pertinent for experienced scholars. Fourth, frameworks and deductive logic are useful for comparative work because some degree of consistency across cases is built into the design.

As we have seen, the hypotheses of explanatory and categories of descriptive research are the dominate frames of social science and policy science. We certainly concur that neither of these frames makes a lot of sense for exploratory research. They would tend to tie it down. We see the problem as a missing framework or missing way to frame deductive, exploratory research in the methodology literature. Inductive exploratory research would not work for many case studies that are trying to use evidence to make an argument. What exploratory deductive case studies need is a framework that incorporates flexibility. This is even more true for comparative case studies. A framework of this sort could be usefully applied to policy research (Casula 2020a), particularly evaluative policy research, and applied research generally. We propose the Working Hypothesis as a flexible conceptual framework and as a useful tool for doing exploratory studies. It can be used as an evaluative criterion particularly for process evaluation and is useful for student research because students can develop theorizing skills using the literature.

Table 1 included a column specifying the philosophical basis for each research purpose. Shifting gears to the philosophical underpinning of methodology provides useful additional context for examination of deductive, exploratory research.

## What is a working hypothesis

The working hypothesis is first and foremost a hypothesis or a statement of expectation that is tested in action. The term “working” suggest that these hypotheses are subject to change, are provisional and the possibility of finding contradictory evidence is real. In addition, a “working” hypothesis is active, it is a tool in an ongoing process of inquiry. If one begins with a research question, the working hypothesis could be viewed as a statement or group of statements that answer the question. It “works” to move purposeful inquiry forward. “Working” also implies some sort of community, mostly we work together in relationship to achieve some goal.

Working Hypothesis is a term found in earlier literature. Indeed, both pioneering pragmatists, John Dewey and George Herbert Mead use the term working hypothesis in important nineteenth century works. For both Dewey and Mead, the notion of a working hypothesis has a self-evident quality and it is applied in a big picture context.^2^

Most notably, Dewey (1896), in one of his most pivotal early works (“Reflex Arc”), used “working hypothesis” to describe a key concept in psychology. “The idea of the reflex arc has upon the whole come nearer to meeting this demand for a *general working hypothesis* than any other single concept (Italics added)” (p. 357). The notion of a working hypothesis was developed more fully 42 years later, in *Logic the Theory of Inquiry*, where Dewey developed the notion of a working hypothesis that operated on a smaller scale. He defines working hypotheses as a “provisional, working means of advancing investigation” (Dewey 1938, pp. 142). Dewey’s definition suggests that working hypotheses would be useful toward the beginning of a research project (e.g., exploratory research).

Mead (1899) used working hypothesis in a title of an *American Journal of Sociology* article “The *Working Hypothesis* and Social Reform” (italics added). He notes that a scientist’s foresight goes beyond testing a hypothesis.

Given its success, he may restate his world from this standpoint and get the basis for further investigation that again always takes the form of a problem. The solution of this problem is found over again in the possibility of fitting his hypothetical proposition into the whole within which it arises. And he must recognize that this statement is only a working hypothesis at the best, i.e., he knows that further investigation will show that the former statement of his world is only provisionally true, and must be false from the standpoint of a larger knowledge, as every partial truth is necessarily false over against the fuller knowledge which he will gain later (Mead 1899, p. 370).

Cronbach (1975) developed a notion of working hypothesis consistent with inductive reasoning, but for him, the working hypothesis is a product or result of naturalistic inquiry. He makes the case that naturalistic inquiry is highly context dependent and therefore results or seeming generalizations that may come from a study and should be viewed as “working hypotheses”, which “are tentative both for the situation in which they first uncovered and for other situations” (as cited in Gobo 2008, p. 196).

A quick Google scholar search using the term “working hypothesis” show that it is widely used in twentieth and twenty-first century science, particularly in titles. In these articles, the working hypothesis is treated as a conceptual tool that furthers investigation in its early or transitioning phases. We could find no explicit links to exploratory research. The exploratory nature of the problem is expressed implicitly. Terms such as “speculative” (Habib 2000, p. 2391) or “rapidly evolving field” (Prater et al. 2007, p. 1141) capture the exploratory nature of the study. The authors might describe how a topic is “new” or reference “change”. “As a working hypothesis, the picture is only new, however, in its interpretation” (Milnes 1974, p. 1731). In a study of soil genesis, Arnold (1965, p. 718) notes “Sequential models, formulated as working hypotheses, are subject to further investigation and change”. Any 2020 article dealing with COVID-19 and respiratory distress would be preliminary almost by definition (Ciceri et al. 2020).

## Philosophical roots of methodology

According to Kaplan (1964, p. 23) “the aim of methodology is to help us understand, in the broadest sense not the products of scientific inquiry but the process itself”. Methods contain philosophical principles that distinguish them from other “human enterprises and interests” (Kaplan 1964, p. 23). Contemporary research methodology is generally classified as quantitative, qualitative and mixed methods. Leading scholars of methodology have associated each with a philosophical underpinning—positivism (or post-positivism), interpretivism or constructivist and pragmatism, respectively (Guba 1987; Guba and Lincoln 1981; Schrag 1992; Stebbins 2001; Mackenzi and Knipe 2006; Atieno 2009; Levers 2013; Morgan 2007; O’Connor et al. 2008; Johnson and Onwuegbuzie 2004; Twining et al. 2017). This section summarizes how the literature often describes these philosophies and informs contemporary methodology and its literature.

Positivism and its more contemporary version, post-positivism, maintains an objectivist ontology or assumes an objective reality, which can be uncovered (Levers 2013; Twining et al. 2017).^3^ Time and context free generalizations are possible and “real causes of social scientific outcomes can be determined reliably and validly (Johnson and Onwuegbunzie 2004, p. 14). Further, “explanation of the social world is possible through a logical reduction of social phenomena to physical terms”. It uses an empiricist epistemology which “implies testability against observation, experimentation, or comparison” (Whetsell and Shields 2015, pp. 420–421). Correspondence theory, a tenet of positivism, asserts that “to each concept there corresponds a set of operations involved in its scientific use” (Kaplan 1964, p. 40).

The interpretivist, constructivists or post-modernist approach is a reaction to positivism. It uses a relativist ontology and a subjectivist epistemology (Levers 2013). In this world of multiple realities, context free generalities are impossible as is the separation of facts and values. Causality, explanation, prediction, experimentation depend on assumptions about the correspondence between concepts and reality, which in the absence of an objective reality is impossible. Empirical research can yield “contextualized emergent understanding rather than the creation of testable theoretical structures” (O’Connor et al. 2008, p. 30). The distinctively different world views of positivist/post positivist and interpretivist philosophy is at the core of many controversies in methodology, social and policy science literature (Casula 2020b).

With its focus on dissolving dualisms, pragmatism steps outside the objective/subjective debate. Instead, it asks, “what difference would it make to us if the statement were true” (Kaplan 1964, p. 42). Its epistemology is connected to *purposeful* inquiry. Pragmatism has a “transformative, experimental notion of inquiry” anchored in pluralism and a focus on constructing conceptual and practical tools to resolve “problematic situations” (Shields 1998; Shields and Rangarajan 2013). Exploration and working hypotheses are most comfortably situated within the pragmatic philosophical perspective.

## Research approaches

Empirical investigation relies on three types of methodology—quantitative, qualitative and mixed methods.

### Quantitative methods

Quantitative methods uses deductive logic and formal hypotheses or models to explain, predict, and eventually establish causation (Hyde 2000; Kaplan 1964; Johnson and Onwuegbunzie 2004; Morgan 2007).^4^ The correspondence between the conceptual and empirical world make measures possible. Measurement assigns numbers to objects, events or situations and allows for standardization and subtle discrimination. It also allows researchers to draw on the power of mathematics and statistics (Kaplan 1964, pp. 172–174). Using the power of inferential statistics, quantitative research employs research designs, which eliminate competing hypotheses. It is high in external validity or the ability to generalize to the whole. The research results are relatively independent of the researcher (Johnson & Onwuegbunzie 2004).

Quantitative methods depend on the quality of measurement and a priori conceptualization, and adherence to the underlying assumptions of inferential statistics. Critics charge that hypotheses and frameworks needlessly constrain inquiry (Johnson and Onwuegbunzie 2004, p. 19). Hypothesis testing quantitative methods support the explanatory purpose.

### Qualitative methods

Qualitative researchers who embrace the post-modern, interpretivist view,^5^ question everything about the nature of quantitative methods (Willis et al. 2007). Rejecting the possibility of objectivity, correspondence between ideas and measures, and the constraints of a priori theorizing they focus on “unique impressions and understandings of events rather than to generalize the findings” (Kolb 2012, p. 85). Characteristics of traditional qualitative research include “induction, discovery, exploration, theory/hypothesis generation and the researcher as the primary ‘instrument’ of data collection” (Johnson and Onwuegbunzie 2004, p. 18). It also concerns itself with forming “unique impressions and understandings of events rather than to generalize findings” (Kolb 2012, p. 85). The data of qualitative methods are generated via interviews, direct observation, focus groups and analysis of written records or artifacts.

Qualitative methods provide for understanding and “description of people’s personal experiences of phenomena”. They enable descriptions of detailed “phenomena as they are situated and embedded in local contexts.” Researchers use naturalistic settings to “study dynamic processes” and explore how participants interpret experiences. Qualitative methods have an inherent flexibility, allowing researchers to respond to changes in the research setting. They are particularly good at narrowing to the particular and on the flipside have limited external validity (Johnson and Onwuegbunzie 2004, p. 20). Instead of specifying a suitable sample size to draw conclusions, qualitative research uses the notion of saturation (Morse 1995).

Saturation is used in grounded theory—a widely used and respected form of qualitative research, and a well-known interpretivist qualitative research method. Introduced by Glaser and Strauss (1967), this “grounded on observation” (Patten and Newhart 2000, p. 27) methodology, focuses on “the creation of emergent understanding” (O’Connor et al. 2008, p. 30). It uses the Constant Comparative method, whereby researchers develop theory from data as they code and analyze at the same time. Data collection, coding and analysis along with theoretical sampling are systematically combined to generate theory (Kolb 2012, p. 83). The qualitative methods discussed here support exploratory research.

A close look at the two philosophies and assumptions of quantitative and qualitative research suggests two contradictory world views. The literature has labeled these contradictory views the *Incompatibility Theory,* which sets up a quantitative versus qualitative tension similar to the seeming separation of art and science or fact and values (Smith 1983a, b; Guba 1987; Smith and Heshusius 1986; Howe 1988). The incompatibility theory does not make sense in practice. Yin (1981, 1992, 2011, 2017), a prominent case study scholar, showcases a deductive research methodology that crosses boundaries using both quantaitive and qualitative evidence when appropriate.

### Mixed methods

Turning the “Incompatibility Theory” on its head, Mixed Methods research “combines elements of qualitative and quantitative research approaches … for the broad purposes of breadth and depth of understanding and corroboration” (Johnson et al. 2007, p. 123). It does this by partnering with philosophical pragmatism.^6^ Pragmatism is productive because “it offers an immediate and useful middle position philosophically and methodologically; it offers a practical and outcome-oriented method of inquiry that is based on action and leads, iteratively, to further action and the elimination of doubt; it offers a method for selecting methodological mixes that can help researchers better answer many of their research questions” (Johnson and Onwuegbunzie 2004, p. 17). What is theory for the pragmatist “any theoretical model is for the pragmatist, *nothing more than a framework through which problems are perceived and subsequently organized*” (Hothersall 2019, p. 5).

Brendel (2009) constructed a simple framework to capture the core elements of pragmatism. Brendel’s four “p”’s—practical, pluralism, participatory and provisional help to show the relevance of pragmatism to mixed methods. Pragmatism is purposeful and concerned with the *practical* consequences. The *pluralism* of pragmatism overcomes quantitative/qualitative dualism. Instead, it allows for multiple perspectives (including positivism and interpretivism) and, thus, gets around the incompatibility problem. Inquiry should be *participatory* or inclusive of the many views of participants, hence, it is consistent with multiple realities and is also tied to the common concern of a problematic situation. Finally, all inquiry is *provisional*. This is compatible with experimental methods, hypothesis testing and consistent with the back and forth of inductive and deductive reasoning. Mixed methods support exploratory research.

Advocates of mixed methods research note that it overcomes the weaknesses and employs the strengths of quantitative and qualitative methods. Quantitative methods provide precision. The pictures and narrative of qualitative techniques add meaning to the numbers. Quantitative analysis can provide a big picture, establish relationships and its results have great generalizability. On the other hand, the “why” behind the explanation is often missing and can be filled in through in-depth interviews. A deeper and more satisfying explanation is possible. Mixed-methods brings the benefits of triangulation or multiple sources of evidence that converge to support a conclusion. It can entertain a “broader and more complete range of research questions” (Johnson and Onwuegbunzie 2004, p. 21) and can move between inductive and deductive methods. Case studies use multiple forms of evidence and are a natural context for mixed methods.

One thing that seems to be missing from mixed method literature and explicit design is a place for conceptual frameworks. For example, Heyvaert et al. (2013) examined nine mixed methods studies and found an explicit framework in only two studies (transformative and pragmatic) (p. 663).

## Theory and hypotheses: where is and what is theory?

Theory is key to deductive research. In essence, empirical deductive methods test theory. Hence, we shift our attention to theory and the role and functions of the hypotheses in theory. Oppenheim and Putnam (1958) note that “by a ‘theory’ (in the widest sense) we mean any hypothesis, generalization or law (whether deterministic or statistical) or any conjunction of these” (p. 25). Van Evera (1997) uses a similar and more complex definition “theories are general statements that describe and explain the causes of effects of classes of phenomena. They are composed of causal laws or hypotheses, explanations, and antecedent conditions” (p. 8). Sutton and Staw (1995, p. 376) in a highly cited article “What Theory is Not” assert the that hypotheses should contain logical arguments for “why” the hypothesis is expected. Hypotheses need an underlying causal argument before they can be considered theory. The point of this discussion is not to define theory but to establish the importance of hypotheses in theory.

Explanatory research is implicitly relational (A explains B). The hypotheses of explanatory research lay bare these relationships. Popular definitions of hypotheses capture this relational component. For example, the *Cambridge Dictionary* defines a hypothesis a “an idea or explanation for something that is based on known facts but has not yet been proven”. *Vocabulary.Com’s* definition emphasizes explanation, a hypothesis is “an idea or explanation that you then test through study and experimentation”. According to *Wikipedia* a hypothesis is “a proposed explanation for a phenomenon”. Other definitions remove the relational or explanatory reference. The *Oxford English Dictionary* defines a hypothesis as a “supposition or conjecture put forth to account for known facts.” *Science Buddies* defines a hypothesis as a “tentative, testable answer to a scientific question”. According to the *Longman Dictionary* the hypothesis is “an idea that can be tested to see if it is true or not”. The *Urban Dictionary* states a hypothesis is “a prediction or educated-guess based on current evidence that is yet be tested”. We argue that the hypotheses of exploratory research—*working hypothesis*—*are not bound by relational expectations. It is this flexibility that distinguishes the working hypothesis.*

Sutton and Staw (1995) maintain that hypotheses “serve as crucial *bridges* between theory and data, making explicit how the variables and relationships that follow from a logical argument will be operationalized” (p. 376, italics added). The highly rated journal, *Computers and Education*, Twining et al. (2017) created guidelines for qualitative research as a way to improve soundness and rigor. They identified the lack of alignment between theoretical stance and methodology as a common problem in qualitative research. In addition, they identified a lack of alignment between methodology, design, instruments of data collection and analysis. The authors created a guidance summary, which emphasized the need to enhance coherence throughout elements of research design (Twining et al. 2017 p. 12). Perhaps the *bridging* function of the hypothesis mentioned by Sutton and Staw (1995) is obscured and often missing in qualitative methods. Working hypotheses can be a tool to overcome this problem.

For reasons, similar to those used by mixed methods scholars, we look to classical pragmatism and the ideas of John Dewey to inform our discussion of theory and working hypotheses. Dewey (1938) treats theory as a tool of empirical inquiry and uses a map metaphor (p. 136). Theory is like a map that helps a traveler navigate the terrain—and should be judged by its usefulness. “There is no expectation that a map is a true representation of reality. Rather, it is a representation that allows a traveler to reach a destination (achieve a purpose). Hence, theories should be judged by how well they help resolve the problem or *achieve a purpose*” (Shields and Rangarajan 2013, p. 23). Note that we explicitly link theory to the research purpose. Theory is never treated as an unimpeachable Truth, rather it is a helpful tool that organizes inquiry connecting data and problem. Dewey’s approach also expands the definition of theory to include abstractions (categories) outside of causation and explanation. The micro-conceptual frameworks^7^ introduced in Table 1 are a type of theory. We define conceptual frameworks as the “way the ideas are organized to achieve the project’s purpose” (Shields and Rangarajan 2013 p. 24). Micro-conceptual frameworks do this at the very close to the data level of analysis. Micro-conceptual frameworks can direct operationalization and ways to assess measurement or evidence at the individual research study level. Again, the research purpose plays a pivotal role in the functioning of theory (Shields and Tajalli 2006).

## Working hypothesis: methods and data analysis

We move on to answer the remaining questions in the Table 1. We have established that exploratory research is extremely flexible and idiosyncratic. Given this, we will proceed with a few examples and draw out lessons for developing an exploratory purpose, building a framework and from there identifying data collection techniques and the logics of hypotheses testing and analysis. Early on we noted the value of the Working Hypothesis framework for student empirical research and applied research. The next section uses a masters level student’s work to illustrate the usefulness of working hypotheses as a way to incorporate the literature and structure inquiry. This graduate student was also a mature professional with a research question that emerged from his job and is thus an example of applied research.

Master of Public Administration student, Swift (2010) worked for a public agency and was responsible for that agency’s sexual harassment training. The agency needed to evaluate its training but had never done so before. He also had never attempted a significant empirical research project. Both of these conditions suggest exploration as a possible approach. He was interested in evaluating the training program and hence the project had a normative sense. Given his job, he already knew a lot about the problem of sexual harassment and sexual harassment training. What he did not know much about was doing empirical research, reviewing the literature or building a framework to evaluate the training (working hypotheses). He wanted a framework that was flexible and comprehensive. In his research, he discovered Lundvall’s (2006) knowledge taxonomy summarized with four simple ways of knowing (*Know*-*what, Know*-*how, Know*-*why, Know*-*who*). He asked whether his agency’s training provided the participants with these kinds of knowledge? Lundvall’s categories of knowing became the basis of his working hypotheses. Lundvall’s knowledge taxonomy is well suited for working hypotheses because it is so simple and is easy to understand intuitively. It can also be tailored to the unique problematic situation of the researcher. Swift (2010, pp. 38–39) developed four basic working hypotheses:

- WH1: Capital Metro provides adequate *know*-*what* knowledge in its sexual harassment training
- WH2: Capital Metro provides adequate *know*-*how* knowledge in its sexual harassment training
- WH3: Capital Metro provides adequate *know*-*why* knowledge in its sexual harassment training
- WH4: Capital Metro provides adequate *know*-*who* knowledge in its sexual harassment training

From here he needed to determine what would determine the different kinds of knowledge. For example, what constitutes “know what” knowledge for sexual harassment training. This is where his knowledge and experience working in the field as well as the literature come into play. According to Lundvall et al. (1988, p. 12) “know what” knowledge is about facts and raw information. Swift (2010) learned through the literature that laws and rules were the basis for the mandated sexual harassment training. He read about specific anti-discrimination laws and the subsequent rules and regulations derived from the laws. These laws and rules used specific definitions and were enacted within a historical context. Laws, rules, definitions and history became the “facts” of Know-What knowledge for his working hypothesis. To make this clear, he created sub-hypotheses that explicitly took these into account. See how Swift (2010, p. 38) constructed the sub-hypotheses below. Each sub-hypothesis was defended using material from the literature (Swift 2010, pp. 22–26). The sub-hypotheses can also be easily tied to evidence. For example, he could document that the training covered anti-discrimination laws.

WH1: Capital Metro provides adequate *know*-*what* knowledge in its sexual Harassment training

- WH1a: The sexual harassment training includes information on anti-discrimination laws (Title VII).
- WH1b: The sexual harassment training includes information on key definitions.
- WH1c: The sexual harassment training includes information on Capital Metro’s Equal Employment Opportunity and Harassment policy.
- WH1d: Capital Metro provides training on sexual harassment history.

Know-How knowledge refers to the ability to do something and involves skills (Lundvall and Johnson 1994, p. 12). It is a kind of expertise in action. The literature and his experience allowed James Smith to identify skills such as how to file a claim or how to document incidents of sexual harassment as important “know-how” knowledge that should be included in sexual harassment training. Again, these were depicted as sub-hypotheses.

WH2: Capital Metro provides adequate *know*-*how* knowledge in its sexual Harassment training

- WH2a: Training is provided on *how to* file and report a claim of harassment
- WH2b: Training is provided on *how to* document sexual harassment situations.
- WH2c: Training is provided on *how to* investigate sexual harassment complaints.
- WH2d: Training is provided on *how to* follow additional harassment policy procedures protocol

**Fig. 1:**
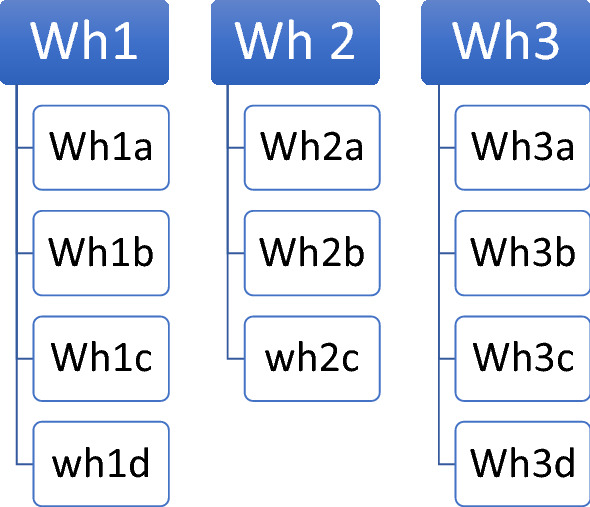
A Common structure used in the development of working hypotheses

Note that the working hypotheses do not specify a relationship but rather are simple declarative sentences. If “know-how” knowledge was found in the sexual harassment training, he would be able to find evidence that participants learned about how to file a claim (WH2a). The working hypothesis provides the bridge between theory and data that Sutton and Staw (1995) found missing in exploratory work. The sub-hypotheses are designed to be refined enough that the researchers would know what to look for and tailor their hunt for evidence. Figure 1 captures the generic sub-hypothesis design.

When expected evidence is linked to the sub-hypotheses, data, framework and research purpose are aligned. This can be laid out in a planning document that *operationalizes* the data collection in something akin to an architect’s blueprint. This is where the scholar explicitly develops the *alignment* between purpose, framework and method (Shields and Rangarajan 2013; Shields et al. 2019b).

Table 2 operationalizes Swift’s working hypotheses (and sub-hypotheses). The table provide clues as to what kind of evidence is needed to determine whether the hypotheses are supported. In this case, Smith used interviews with participants and trainers as well as a review of program documents. Column one repeats the sub-hypothesis, column two specifies the data collection method (here interviews with participants/managers and review of program documents) and column three specifies the unique questions that focus the investigation. For example, the interview questions are provided. In the less precise world of qualitative data, evidence supporting a hypothesis could have varying degrees of strength. This too can be specified.

**Table 2:** Operationalization of the working hypotheses: an example

Working hypothesis	Method of data collection	Evidence/criteria
*WH1: Capital Metro provides adequate know*-*what knowledge in its sexual Harassment training*		
WH1a: Capital Metro provides training on anti-discrimination laws (Title VII)	Interview supervisors and managers	(Interview questions) How does the sexual harassment training address anti-discrimination laws? Additional questions as appropriate
	Interview participants	(Interview questions) What did you learn about anti-discrimination law? Additional questions as appropriate
	Document analysis (1) Capital Metro EEO Basic training manual (2) Capital Metro EEO Policy	How do these documents address history of discrimination laws? Additional questions as appropriate
WH1b: Capital Metro provides training on sexual harassment definitions	Interview supervisors and managers	(Interview questions) How does the sexual harassment training address key definitions? Additional questions as appropriate
	Interview participants	(Interview questions) What definitions about sexual harassment did you learn? Additional questions as appropriate
	Document Analysis (1) Capital Metro EEO Basic training manual (2) Capital Metro EEO Policy	Which definitions can be found in these documents?
WH1c: and WH1d	Data collection methods	Further specification of evidence used
*WH2: Capital Metro sexual harassment training provides adequate “know how” training*		
WH2a: Capital Metro provides training on *how*-*to* file and report a claim of harassment	Interview supervisors and managers	(Interview questions) How well does the sexual harassment training prepare participants to file and report a claim? Additional questions as appropriate
	Interview with participants	(Interview questions) How well did the sexual harassment training prepare you to file and report a claim? Additional questions as appropriate
	Document analysis (1) Capital Metro EEO Basic training manual (2) Capital Metro EEO Policy	How well do the documents cover how-to file a report of sexual harassment?
WH2b: Capital Metro provides training on *how to* document sexual harassment situations	Interview of supervisors and managers	(Interview questions) How does the sexual harassment training address how to document sexual harassment situations? Additional questions as appropriate
	Interview participants	
	(Interview questions) How well did the training cover how to document sexual harassment situations? Additional questions as appropriate	
	Document analysis (1) Capital Metro EEO Basic training manual (2) Capital Metro EEO Policy	How well do these documents address procedures to document sexual harassment situations
Wh2c & d	Data collection methods. Etc.	Specify further evidence used

For Swift’s example, neither the statistics of explanatory research nor the open-ended questions of interpretivist, inductive exploratory research is used. The deductive logic of inquiry here is somewhat intuitive and similar to a detective (Ulriksen and Dadalauri 2016). It is also a logic used in international law (Worster 2013). It should be noted that the working hypothesis and the corresponding data collection protocol does not stop inquiry and fieldwork outside the framework. The interviews could reveal an unexpected problem with Smith’s training program. The framework provides a very loose and perhaps useful ways to identify and make sense of the data that does not fit the expectations. Researchers using working hypotheses should be sensitive to interesting findings that fall outside their framework. These could be used in future studies, to refine theory or even in this case provide suggestions to improve sexual harassment training. The sensitizing concepts mentioned by Gilgun (2015) are free to emerge and should be encouraged.

Something akin to working hypotheses are hidden in plain sight in the professional literature. Take for example Kerry Crawford’s (2017) book *Wartime Sexual Violence.* Here she explores how basic changes in the way “advocates and decision makers think about and discuss conflict-related sexual violence” (p. 2). She focused on a subsequent shift from silence to action. The shift occurred as wartime sexual violence was reframed as a “weapon of war”. The new frame captured the attention of powerful members of the security community who demanded, initiated, and paid for institutional and policy change. Crawford (2017) examines the legacy of this key reframing. She develops a six-stage model of potential international responses to incidents of wartime violence. This model is fairly easily converted to working hypotheses and sub-hypotheses. Table 3 shows her model as a set of (non-relational) working hypotheses. She applied this model as a way to gather evidence among cases (e.g., the US response to sexual violence in the Democratic Republic of the Congo) to show the official level of response to sexual violence. Each case study chapter examined evidence to establish whether the case fit the pattern formalized in the working hypotheses. The framework was very useful in her comparative context. The framework allowed for consistent comparative analysis across cases. Her analysis of the three cases went well beyond the material covered in the framework. She freely incorporated useful inductively informed data in her analysis and discussion. The framework, however, allowed for alignment within and across cases.

**Table 3:** Example illustrating a set of working hypotheses as a framework for comparative case studies *Source*: Adaptation from Table 1.1 of Crawford’s (2017) book *Wartime Sexual Violence*

Stages of potential international response to sexual violence
*WH1: Nonrecognition or no action to prevent sexual violence during conflict*
WH1a: Sexual violence is not recognized as part of a specific conflict or the conflict itself is not recognized
WH1b: Wartime sexual violence as a general issue is not recognized
WH1c: No action is taken, and no formal discussion occurs within or among International Organizations (IO)
*WH2: Sexual Violence is documented during a conflict and learning occurs*
WH2a: Sexual violence as an aspect of a conflict is the subject of a report, publication, study or conference attended by a state or IO
WH2b: Information gathering about sexual violence during a conflict occurs
*H3: There is a rhetorical response and condemnation of sexual violence during a conflict*
WH3a: Sexual violence as part of a specific conflict is subject of a speech, unprompted remarks or press release of a high-ranking state official or leader of an IO
WH3b: Rhetorical remarks occur but resources to reduce or study sexual violence are not committed
Three additional stages of international response were provided by Crawford (2017)

## Conclusion

In this article we argued that the exploratory research is also well suited for deductive approaches. By examining the landscape of deductive, exploratory research, we proposed the working hypothesis as a flexible conceptual framework and a useful tool for doing exploratory studies. It has the potential to guide and bring coherence across the steps in the research process. After presenting the nature of exploratory research purpose and how it differs from two types of research purposes identified in the literature—explanation, and description. We focused on answering four different questions in order to show the link between micro-conceptual frameworks and research purposes in a deductive setting. The answers to the four questions are summarized in Table 4.

**Table 4:** Linking micro-conceptual frameworks and research purposes in deductive research


Type of purpose	Micro-conceptual framework	Methodology	Data analysis	Primary philosophical underpinning
Explanatory	Formal Hypotheses	Quantitative, experimental design, survey, time series, existing data	Inferential statistics	Positivism
Descriptive	Categories	Quantitative, survey, content analysis	Simple descriptive statistics	Positivism
**Exploratory**	**Working hypotheses**	**Qualitative, mixed methods, case study, quantitative**	**Evidence of all types may or may not use statistics**	**Pragmatism**

Firstly, we argued that working hypothesis and exploration are situated within the pragmatic philosophical perspective. Pragmatism allows for pluralism in theory and data collection techniques, which is compatible with the flexible exploratory purpose. Secondly, after introducing and discussing the four core elements of pragmatism (practical, pluralism, participatory, and provisional), we explained how the working hypothesis informs the methodologies and evidence collection of deductive exploratory research through a presentation of the benefits of triangulation provided by mixed methods research. Thirdly, as is clear from the article title, we introduced the working hypothesis as the micro-conceptual framework for deductive explorative research. We argued that the hypotheses of explorative research, which we call working hypotheses are distinguished from those of the explanatory research, since they do not require a relational component and are not bound by relational expectations. A working hypothesis is extremely flexible and idiosyncratic, and it could be viewed as a statement or group of statements of expectations tested in action depending on the research question. Using examples, we concluded by explaining how working hypotheses inform data collection and analysis for deductive exploratory research.

Crawford’s (2017) example showed how the structure of working hypotheses provide a framework for comparative case studies. Her criteria for analysis were specified ahead of time and used to frame each case. Thus, her comparisons were systemized across cases. Further, the framework ensured a connection between the data analysis and the literature review. Yet the flexible, working nature of the hypotheses allowed for unexpected findings to be discovered.

The evidence required to test working hypotheses is directed by the research purpose and potentially includes both quantitative and qualitative sources. Thus, all types of evidence, including quantitative methods should be part of the toolbox of deductive, explorative research. We show how the working hypotheses, as a flexible exploratory framework, resolves many seeming dualisms pervasive in the research methods literature.

To conclude, this article has provided an in-depth examination of working hypotheses taking into account philosophical questions and the larger formal research methods literature. By discussing working hypotheses as applied, theoretical tools, we demonstrated that working hypotheses fill a unique niche in the methods literature, since they provide a way to enhance alignment in deductive, explorative studies.

## Footnotes

1 In practice, quantitative scholars often run multivariate analysis on data bases to find out if there are correlations. Hypotheses are tested because the statistical software does the math, not because the scholar has an a priori, relational expectation (hypothesis) well-grounded in the literature and supported by cogent arguments. Hunches are just fine. This is clearly an inductive approach to research and part of the large process of inquiry.

2 In 1958, Philosophers of Science, Oppenheim and Putnam use the notion of Working Hypothesis in their title “Unity of Science as Working Hypothesis.” They too, use it as a big picture concept, “unity of science in this sense, can be fully realized constitutes an over-arching meta-scientific hypothesis, which enables one to see a unity in scientific activities that might otherwise appear disconnected or unrelated” (p. 4).

3 It should be noted that the positivism described in the research methods literature does not resemble philosophical positivism as developed by philosophers like Comte (Whetsell and Shields 2015). In the research methods literature “positivism means different things to different people….The term has long been emptied of any precise denotation …and is sometimes affixed to positions actually opposed to those espoused by the philosophers from whom the name derives” (Schrag 1992, p. 5). For purposes of this paper, we are capturing a few essential ways positivism is presented in the research methods literature. This helps us to position the “working hypothesis” and “exploratory” research within the larger context in contemporary research methods. We are not arguing that the positivism presented here is anything more. The incompatibility theory discussed later, is an outgrowth of this research methods literature…

4 It should be noted that quantitative researchers often use inductive reasoning. They do this with existing data sets when they run correlations or regression analysis as a way to find relationships. They ask, what does the data tell us?

5 Qualitative researchers are also associated with phenomenology, hermeneutics, naturalistic inquiry and constructivism.

6 See Feilzer (2010), Howe (1988), Johnson and Onwuegbunzie (2004), Morgan (2007), Onwuegbuzie and Leech (2005), Biddle and Schafft (2015).

7 The term conceptual framework is applicable in a broad context (see Ravitch and Riggan 2012). The micro-conceptual framework narrows to the specific study and informs data collection (Shields and Rangarajan 2013; Shields et al. 2019a) .
